# Spinal Accessory Nerve Duplication: A Case Report and Literature Review

**DOI:** 10.1155/2018/1027831

**Published:** 2018-04-01

**Authors:** Eleni Papagianni, Panagiota Kosmidou, Sotiria Fergadaki, Athanasios Pallantzas, Panagiotis Skandalakis, Dimitrios Filippou

**Affiliations:** ^1^Department of Otolaryngology-Head and Neck Surgery, 417 Army Share Fund Hospital, Athens, Greece; ^2^Department of Anatomy and Surgical Anatomy, Medical School, National and Kapodistrian University of Athens, Athens, Greece; ^3^Department of Otolaryngology-Head and Neck Surgery, Evaggelismos General Hospital, Athens, Greece; ^4^Plastic and Reconstructive Surgery Department, Evaggelismos General Hospital, Athens, Greece

## Abstract

Aim of the present study is to expand our knowledge of the anatomy of the 11th cranial nerve and discuss the clinical importance and literature pertaining to accessory nerve duplication. We present one case of duplicated spinal accessory nerve in a patient undergoing neck dissection for oral cavity cancer. The literature review confirms the extremely rare diagnosis of a duplicated accessory nerve. Its clinical implication is of great importance. From this finding, a further extension to our knowledge of the existing anatomy is proposed.

## 1. Introduction

Cervical lymphadenectomy or neck dissection is a commonly performed procedure concerning the management of head and neck cancer. A major complication associated with this surgery constitutes the injury to the spinal accessory nerve in addition to injury to the great vessels and lymphatic vessels [1]. Successful surgical management of these patients consequently depends on recognizing the anatomic structures that may potentially put a positive outcome at risk.

The spinal accessory nerve (SAN) is unique in that it shares its innervation from both the medulla and the spinal cord. The cranial fibers, which originate from the nucleus ambiguus in the medulla, are designated to innervate the laryngeal muscles. The spinal component is composed of fibers from the anterior horn cells of the upper five or six cervical vertebrae. These spinal fibers enter the posterior cranial fossa via the foramen magnum, merge with the cranial fibers, and then exit through the jugular foramen along with the fibers of the glossopharyngeal and vagus nerves. The SAN branches off passing deep into the posterior belly of the digastric muscle to supply the sternocleidomastoid. Finally, it transverses the posterior triangle of the neck, superficial to the prevertebral fascia, and terminates with its branches in the deep surface of the trapezius muscle. Some studies have also documented innervation of the upper trapezius directly from the cervical plexus [2].

Spinal accessory nerve can be a cause of scapular winging and shoulder dysfunction. Despite the use of nerve-sparing surgery, the frequency of shoulder morbidity after modified radical or selective neck dissection remains high. A high incidence of shoulder morbidity may be because of the unrecognized anatomical patterns and branching of CN XI. The most common etiology by far is iatrogenic damage (75%) secondary to surgical procedures involving the posterior triangle of the neck. These are often recognized perioperatively. Understanding anatomical variations of the spinal accessory nerve is therefore of crucial importance to avoid iatrogenic injury [2, 3].

We report a single case of duplicated SAN on the left side in a patient undergoing neck dissection and discuss the clinical implications, management, and literature pertaining to SAN duplication.

## 2. Case Report

**Figure 1:**
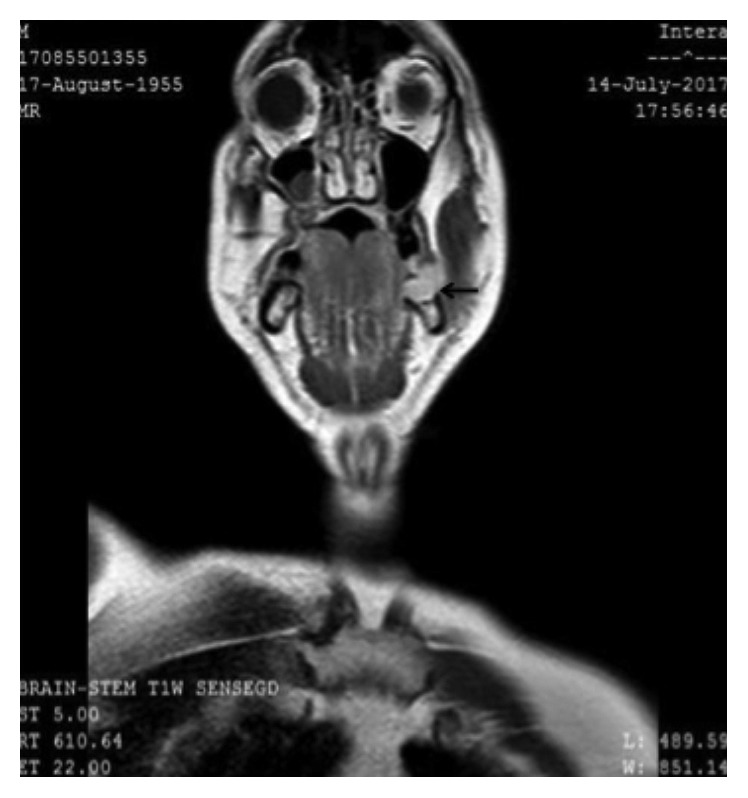
Imaging of the lesion in the internal surface of the mandible on the left in cranial MRI T1 with gadolinium (black arrow).

**Figure 2:**
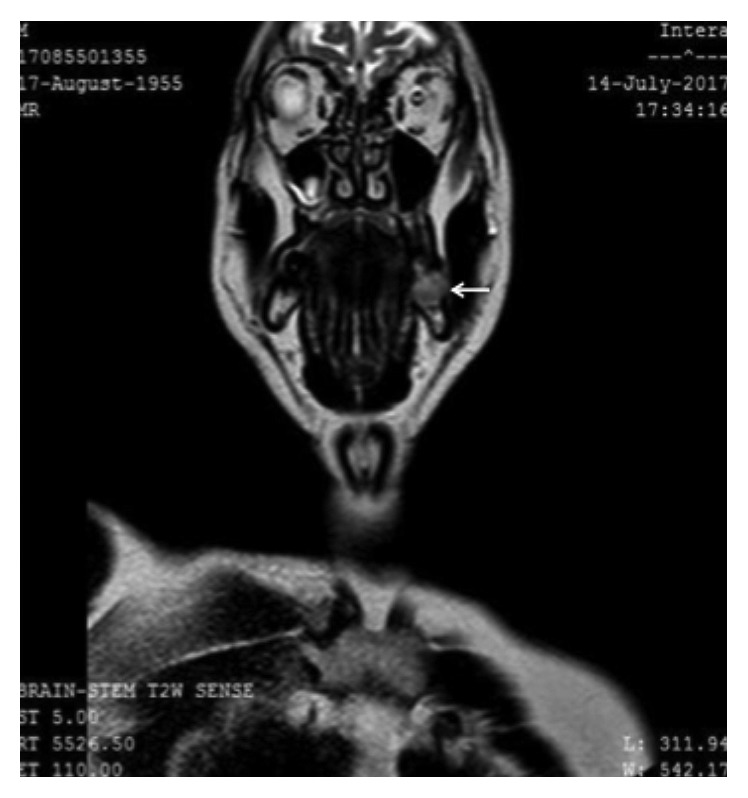
Imaging of the lesion in the internal surface of the mandible on the left in cranial MRI T2 (white arrow).

A 62-year-old Caucasian male presented at the Otolaryngology-Head and Neck Surgery Department of the 417 Army Share Fund Hospital of Athens with an abnormal lesion of the oral cavity on the left side. The lesion was observed one year ago after a dental surgery was performed. It appeared as a painful, nonhealing ulcer, which displayed gradual growth. Regarding his medical history, he suffered from arterial hypertension, managed with angiotensin II receptor blocker. He had no known allergies and used to be a heavy smoker ten years ago (30 pack-years) and a social drinker. Biopsy of the lesion showed squamous cell carcinoma of the internal surface of the posterior third of the body of the mandible, clinically staged as T3N2bM0 after cranial and cervical MRI (Figures 1 and 2) and thoracic CT. At the time of his appearance at our department, the patient had undergone three courses of chemotherapy for a less invasive surgery to be performed. After chemotherapy, his work up included a reassessment with PET/CT and cranial and cervical MRI, which showed a significant reduction of the initial mass in the primary site and the cervical lymphnodes.

**Figure 3:**
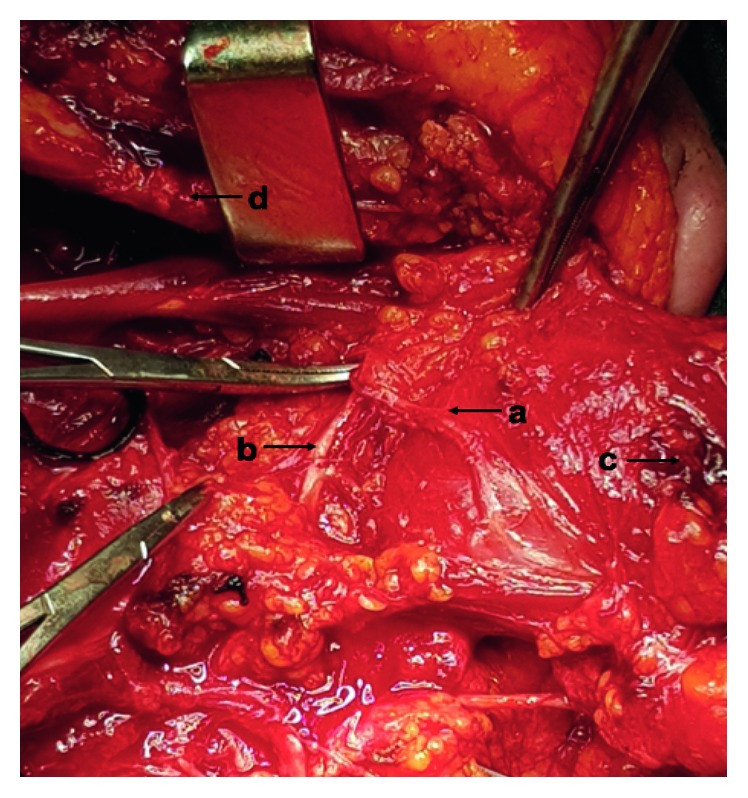
Image captured from left modified radical neck dissection, showing a duplicated spinal accessory nerve. (a) First branch of CN XI. (b) Second branch of CN XI. (c) SCM muscle. and (d) Mandible.

Following this, the patient underwent resection of the primary mass on the mandible and modified radical neck dissection of the levels II to V preserving CN XI. Intraoperatively, a duplication of the spinal accessory nerve was observed, 2 cm inferiorly to the mandible (Figure 3). The first branch was identified penetrating the sternocleidomastoid muscle and the secondary branch fusing with the cervical plexus. The patient recovered from the procedure with minimal regional pain and no evidence of SAN dysfunction.

## 3. Discussion

Spinal accessory nerve duplication is an extremely rare phenomenon. To our knowledge, there have been no reported cases of spinal CN XI duplication. However, one anatomical study on fifty-six cadavers (112 sides) has documented two cases (1.8%) where the nerve was found duplicated, one intracranially and one extracranially [4]. Knowledge of the variants of accessory nerve anatomy may be critical to avoid iatrogenic surgery since SAN is the second most commonly affected peripheral nerve (18%) during surgery in a study, following the median nerve (21.3%) [5].

SAN injury results in loss of motor function of the trapezius muscle and primarily leads to shoulder dysfunction and shoulder pain. The shoulder syndrome after neck dissection was first described by Erwing and Martin in 1952, and it includes constant shoulder pain, shoulder tilt and drop, winged scapula, difficulty of shoulder retraction, restriction in anterior flexion movement and active abduction movements at shoulder joint, and abnormal electromyography findings [6].

The type of neck dissection has major impact on the shoulder dysfunction. Modified radical neck dissection (MRND) increases shoulder morbidity when compared to Selective neck dissection. The frequency of postoperative morbidity of SAN was 46.7% for radical neck dissection, 42.5% for selective neck dissection, and 25% for modified neck dissection in a study. Modified neck dissection has similar regional control rates to more comprehensive operations in appropriately selected patients and significantly reduces the risk of functional disability [7]. Additionally, technique of neck dissection has significant impact on shoulder dysfunction in postoperative period. Contemporary findings regarding the anatomical patterns of CN XI and cervical nerve innervation of the trapezius muscle could have implications for the development of a modified radical neck dissection (MRND) technique [3, 6].

As the exact role of cervical nerve innervation of the trapezius muscle is still unclear, the nerves should be preserved whenever possible [3]. The safest identification of SAN is in the posterior neck triangle where it may be recognized exiting from the posterior border of the sternocleidomastoid muscle at Erb's point [7]. Should the SAN be seriously damaged (transected or a nonrecovering neuroma-in-continuity is observed), treatment would include a primary end-to-end or graft repair. Furthermore, nerve transfers, such as the lateral pectoral nerve, may be considered in cases of proximal injury or delayed presentation [8].

## 4. Conclusion

The identification of anatomical variations of CN XI branching in the neck provides invaluable information to surgeons seeking to preserve the motor branches of the nerve during neck dissection.

## References

[1] Contrera K. J., Aygun N., Ward B. K., Gooi Z., Richmon J. D. (2016). Internal jugular vein duplication and fenestration: case series and literature review. *Laryngoscope*.

[2] Macaluso S., Ross D. C., Doherty T. J., Doherty C. D., Miller T. A. (2016). Spinal accessory nerve injury: a potentially missed cause of a painful, droopy shoulder. *Journal of Back and Musculoskeletal Rehabilitation*.

[3] Lanišnik B. (2017). Different branching patterns of the spinal accessory nerve: impact on neck dissection technique and postoperative shoulder function. *Current Opinion in Otolaryngology & Head and Neck Surgery*.

[4] Tubbs R. S., Ajayi O. O., Fries F. N., Spinner R. J., Oskouian R. J. (2017). Variations of the accessory nerve: anatomical study including previously undocumented findings-expanding our misunderstanding of this nerve. *British Journal of Neurosurgery*.

[5] Rasulić L., Savić A., Vitošević F. (2017). Iatrogenic peripheral nerve injuries—Surgical treatment and outcome: 10 years’ experience. *World Neurosurgery*.

[6] Mathialagan A., Verma R. K., Panda N. K. (2016). Comparison of spinal accessory dysfunction following neck dissection with harmonic scalpel and electrocautery—A randomized study. *Oral Oncology*.

[7] Popovski V., Benedetti A., Popovic-Monevska D., Grcev A., Stamatoski A., Zhivadinovik J. (2017). Head and neck spinal accessory nerve preservation in modified neck dissections: surgical and functional outcomes outcomes. *Acta Otorhinolaryngologica Italica*.

[8] Maldonado A. A., Spinner R. J. (2017). Lateral pectoral nerve transfer for spinal accessory nerve injury. *Journal of Neurosurgery: Spine*.

